# Roles of MALAT1 in development and migration of triple negative and Her-2 positive breast cancer

**DOI:** 10.18632/oncotarget.23370

**Published:** 2017-12-18

**Authors:** Zhang Xiping, Chen Bo, Yang Shifeng, Yu Feijiang, Yang Hongjian, Cheng Qihui, Tang Binbin

**Affiliations:** ^1^ Department of Breast Surgery, Zhejiang Cancer Hospital, Hangzhou 310022, Zhejiang Province, China; ^2^ Department of Pathology, Zhejiang Cancer Hospital, Hangzhou 310022, Zhejiang Province, China; ^3^ Department of Medical Records Room, Zhejiang Cancer Hospital, Hangzhou 310022, Zhejiang Province, China; ^4^ Department of Obstetrics and Gynecology, Hangzhou First People's Hospital, Hangzhou 310006, Zhejiang Province, China; ^5^ Second Outpatient Department of Traditional Chinese Internal Medicine, Tongde Hospital of Zhejiang Province, Hangzhou 310012, Zhejiang Province, China

**Keywords:** breast cancer, MALAT1, triple negative, Her-2 positive

## Abstract

**Background:**

As a type of new targets for prognosis of malignancies, long non-coding RNA MALAT1 (metastasis-associated lung adenocarcinoma transcription 1) is associated with proliferation and metastatic abilities of several malignancies. However, its relations to development and migration of triple negative and human epidermal growth factor receptor 2 (Her-2) positive breast cancers haven't been reported.

**Objectives:**

In this paper, we aimed to discuss how MALAT1 is connected with and affects proliferation and invasion abilities of cells in Her-2 positive and triple-negative breast cancers (TNBC).

**Methods:**

The expression of MALAT1 in clinical samples with TNBC and Her-2 positive breast cancers was tested by qRT-PCR. The statistical analysis was performed to unveil the potential relationships between the expression of MALAT1 and prognostic factors of breast cancer such as OS (overall survival), RFS (relapse-free survival), number of metastatic lymph nodes and pTNM staging in patients with TNBC or Her-2 positive breast cancer. MALAT1 and XBP1 were knockdown respectively in Her-2 positive cell line MDA-MB-231, and MALAT1 and Her-2 were knockdown respectively in TNBC cell line MDA-MD-435 using siRNA. The alterations of expressions of MALAT1 and related genes were detected by qRT-PCR in two breast cancer cell lines. The changes of proliferation abilities in two cell lines were observed using CCK8 assays. Furthermore, transwell assays were performed to detect changes to invasion abilities of the cells.

**Results:**

The expression of MALAT1 in triple negative and Her-2 positive breast cancers was positively correlated to the number of metastatic lymph nodes in patients with breast cancer. MALAT1 promotes proliferation and invasion abilities of breast cancer cells through XBP1 (X-box binding protein 1)-HIF (hypoxia-inducible factor)-1α pathway in MDA-MB-231 and through Her-2 pathway in MDA-MD-435. Moreover, MALAT1 could possibly be involved in regulation of MYC gene and CD47 (an immune checkpoint gene) in both cell lines.

**Conclusions:**

Our study suggested that MALAT1 is a core signaling molecule for promoting development and migration of triple negative and Her-2 positive breast cancers. It would be employed as common markers for prognosis of the two types of breast cancer mentioned above and potential targets for treating them.

## INTRODUCTION

Breast cancer is one of the most common malignancies for females, with the highest incidence among female malignancies in China and even around the world [1]. In recent years, China has become one of countries where the incidence of breast cancer is persistently increasing [2]. An important feature of breast cancer is its high heterogeneity in therapeutic sensitivity, prognosis, metastasis and recurrence, so the needs of most patients with breast cancer cannot be simply satisfied by any kind of singular diagnostic and therapeutic schedule. With the advance of molecular biology, researchers have realized that the differences in the patterns of gene expression would be potential causes of heterogeneity in breast cancer. Targeted molecular therapies are therapeutic schedules for oncogenes of the disease and related expression products, which aim to improve treatment precision of breast cancer. In 2011, experts reached a consensus at the St Gallen International Breast Cancer Conference that breast cancer should be classified into Luminal A, Luminal B, Her-2 positive and triple negative molecular subtypes, in order to develop pertinent clinical therapeutic regimes [3]. Compared with other subtypes, triple-negative breast cancer (TNBC) and Her-2 positive breast cancer are characterized by higher degree of malignancy, higher invasiveness, faster progression, higher recurrence, distant metastasis, poor prognosis and higher mortality. At present, there is still lack of effective therapy for above two subtypes of breast cancer. Although trastuzumab combined with chemotherapy brings survival benefits to patients with Her-2 positive breast cancer, it still has some deficiencies such as drug resistance and costliness [4]. Moreover, there are scarcely any effective targets for treating TNBC. Hence, it is of great significance for further study of molecular mechanisms related to TNBC and Her-2 positive breast cancers, and for the attempt to finding new molecular targets for treating these two kinds of breast cancer.

Long non-coding RNAs (LncRNAs) are non-coding RNA molecules with the length of more than 200 nucleotides, regulating physiologic functions of organisms from the epigenetic perspective, the transcriptional perspective and the post-transcriptional perspective [5]. MALAT1 is a highly conserved lncRNA that is highly expressed in several types of cancer, including breast cancer. It has been demonstrated by *in vivo* and *in vitro* studies that MALAT1 promotes proliferation, tumor development and metastasis of TNBC [6]. In addition, the expression of MALAT1 has been reported to be negatively correlated to the survival of ER negative, lymph node negative patients of the Her-2 and TNBC molecular subtypes [7]. It is particularly noteworthy that a recent study using genetic interventions with MALAT1 antisense nucleotides has achieved good effects for suppressing cancer development in mouse models with luminal B breast cancer [8]. These studies suggested that MALAT1 is expected to become a new biomarker for prognosis of breast cancers and a potential target for treating them.

Nevertheless, there are few studies about the mechanisms by which MALAT1 is involved in the progression of breast cancer, especially breast cancer with different molecular subtypes. Some studies suggested that MALAT1 can promote the progression in several kinds of cancers through different mechanisms, including activating downstream PI3K/AKT [9], regulating transcription [10] and alternative splicing [11], and stimulating EMT of tumors [12]. The highly expressed Her-2 in Her-2 positive breast cancer is a kind of receptor tyrosine kinase (RTK), which mainly activates downstream signaling pathways such as PI3K-Akt and RAS-MAPK through heterogeneous dimerization with other kinds of RTK [13]. According to the facts that have been previously reported, we speculated that MALAT1 and Her-2 have common downstream signaling pathways (including PI3K-Akt [9] and RAS-MAPK [14]), and could interact with each other to regulate progression of Her-2 positive breast cancer.

In TNBC, the expressions of ER, PR and Her-2 are low, and mechainsms of tumor progression in this kind of cancer remain unclear. Recently, it was suggested that under anaerobic conditions, the XBP1-HIF-1α pathway plays important roles in promoting progression of TNBC [15]. In addition, it has been reported that anoxia may activate the transcription of MALAT1 by upregulating HIF1α [16]. Thus, the mechanism for MALAT1 to promote the progression of TNBC could be associated with XBP1-HIF-1α.

Over the past years, immunotherapies have become novel ways for treating malignancies other than surgeries, radiotherapies and chemotherapies. Cancer cells regulate the expression levels of immune checkpoints and inhibit killing effects of host immune systems, so genes regarding the immune checkpoints, including PD-L1 [17] and CD47 [18] have become potential targets for cancer immunotherapies. It has been reported that MALAT1 activates Wnt/β-catenin and the expression of downstream c-Myc through transcriptional regulation [10]. Moreover, MYC activates expressions of CD47 and PD-L1 in cancer cells [19]. Therefore, we speculated that MALAT1 could impact cancer progression and metastasis by regulating activity of immune checkpoint genes.

In this paper, we discussed how the expression level of MALAT1 was associated with metastasis of breast cancer and prognosis of patients. The results suggested that MALAT1 showed varying expression levels in different subtypes of breast cancer, which was closely associated with metastasis of breast cancer and patients’ prognosis. To study mechanism of action of MALAT1 in different molecular subtypes of breast cancer, MALAT1 and XBP1 were knockdown respectively in TNBC cell line MDA-MB-231 and MALAT1 and Her-2 were knockdown respectively in Her-2 positive cell line MDA-MD-435. Our results demonstrated that MALAT1 promotes proliferation and invasion abilities of both malignant tumor cells through different molecular mechanisms. Altogether, we suggested that MALAT1 would become a common biomarker for prognosis of triple negative and Her-2 positive breast cancer. It could also be developed into a potential target for treating these two kinds of breast cancer.

## RESULTS

### Analysis of correlations between expressions of MALAT1 and prognosis of breast cancer in samples with triple negative and Her-2 positive breast cancers

To explore whether the expressions of MALAT1 in triple negative and Her-2 positive breast cancers were correlated to patients’ prognostic factors, preliminary experiments were designed and the expressions of MALAT1 in samples were detected by qRT-PCR. Furthermore, how the expression levels were correlated to different prognostic factors such as patients’ age, tumor size, staging of lymph nodes, pTNM staging, overall survival and relapse-free survival was examined, as shown in Table 1. The results of the analysis suggested that the expression levels of MALAT1 were negatively correlated to OS of patients with triple negative and Her-2 positive patients, but the correlations were not significant. Although they had significant negative correlations with RFS of Her-2 positive patients, they were insignificantly correlated to the RFS of patients with TNBC. They were positively correlated to degree of lymph node metastasis (staging of lymph nodes) and degree of malignancy in patients with triple negative and Her-2 positive breast cancers (Table 1). In addition, a multivariate analysis was performed on prognostic factors which were significantly correlated, including RFS, staging of lymph nodes and pTNM staging. According to the results, the expression of MALAT1 was an independent prognostic factor for metastasis of lymph nodes (staging of lymph nodes) (Table 2). Hence, high expressions of MALAT1 were closely related to metastasis and relapse of both kinds of breast cancer.

**Table 1:** Correlation Analysis between MALAT1 expression level and prognostic factors of samples of triple negative and Her-2 positive breast cancer patients

	Correlation analysis of prognostic factors of triple negative breast cancer patients			Correlation analysis of prognostic factors of Her-2 positive breast cancer patients		
	95% CI	R value (Pearson correlation coefficient)	P value	95% CI	R value (Pearson correlation coefficient)	P value
OS	-0.7193 to 0.5195	-0.1638	0.651	-0.8985 to 0.01743	-0.6191	0.056
RFS	-0.7773 to 0.4164	-0.2891	0.418	-0.9290 to -0.1672	-0.7210	0.019
Tumor size	-0.2576 to 0.8392	0.4442	0.199	-0.6115 to 0.6473	0.0296	0.935
Lymph node staging	0.6331 to 0.9771	0.9029	0.000	0.8968 to 0.9944	0.9756	0.000
PTNM staging	0.06122 to 0.9127	0.6653	0.036	0.6592 to 0.9790	0.9108	0.000
Age	-0.6678 to 0.5884	-0.0657	0.857	-0.5196 to 0.7193	0.1637	0.6514

**Table 2:** Multivariate analysis between MALAT1 expression level and prognostic factors of samples of triple negative and Her-2 positive breast cancer patients

	Multivariate analysis of triple negative breast cancer patients		Multivariate analysis of Her-2 positive breast cancer patients	
	B value (standard partial regression coefficient)	P value	B value (standard partial regression coefficient)	P value
RFS	-	-	-0.126	0.253
Lymph node staging	1.876	0.000	0.976	0.000
pTNM staging	-1.109	0.000	-0.067	0.793

### Analysis on how metastasis of breast cancer is associated with MALAT1 and expression levels of related genes in samples with triple negative and Her-2 positive breast cancers

**Figure 1:**
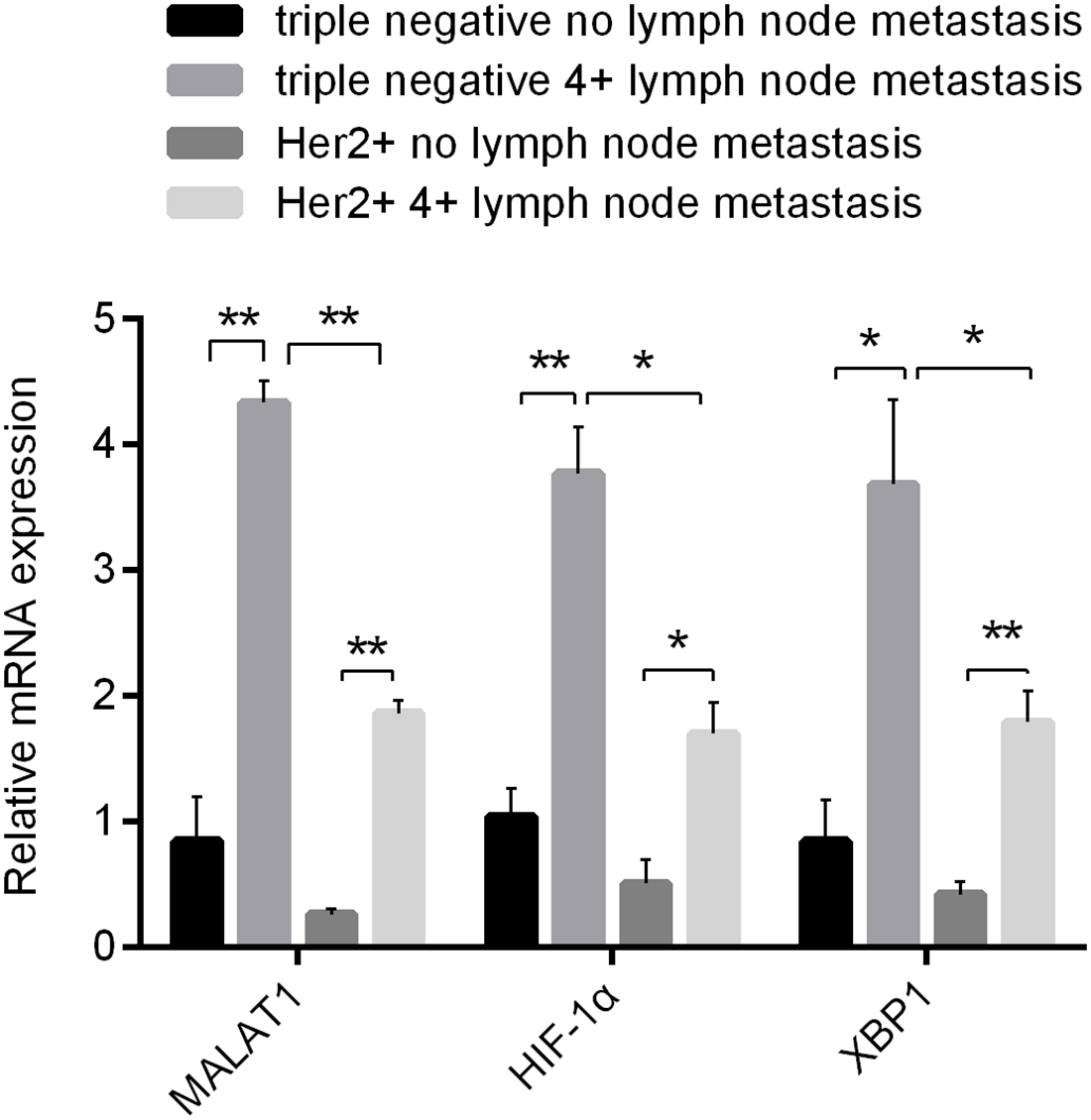
Differences in Expressions of MALAT1, HIF-1α and XBP1 in 2 Kinds of Breast Cancer without and with Metastatic Lymph Nodes (≥4) Detected by qRT-PCR ^*^, p<0.05; ^**^, p<0.01.

High metastasis of triple negative and Her-2 positive breast cancers is an important cause of poor prognosis. To study correlations between MALAT1 and metastatic capacity of two different molecular subtypes of breast cancer, the expressions of MALAT1 in samples with non-metastatic TNBC, highly metastatic TNBC (no fewer than 4 metastatic lymph nodes), non-metastatic, and highly metastatic Her-2 positive breast cancer (no fewer than 4 metastatic lymph nodes) were detected by qRT-PCR. As shown in Figure 1, the expressions of MALAT1 were significantly higher in samples with TNBC than those in samples with Her-2 positive breast cancer. Among samples with Her-2 positive breast cancer, the expressions of MALAT1 were significantly higher in highly metastatic samples compared with non-metastatic samples. Likewise, among samples with TNBC, the expressions of MALAT1 were significatnly higher in highly metastatic samples than those in non-metastatic samples. These results suggested that the expressions of MALAT1 were closely associated with metastatic capacity of breast cancer, whereas the mechanism of MALAT1 for promoting metastasis might be different between triple negative and Her-2 positive breast cancer.

To further investigate potential molecular mechanisms of MALAT1 for promoting metastasis of triple negative and Her-2 positive breast cancers, the expressions of XBP1 and HIF-1α in samples with non-metastatic TNBC, highly metastatic TNBC, non-metastatic Her-2 positive and highly metastatic Her-2 positive breast cancer were detected by qRT-PCR. Among samples with TNBC, XBP1 and HIF-1α exhibited much higher expressions in samples with highly metastatic breast cancer than those with poor metastasis (Figure 1). Likewise, their expressions were positively correlated to metastasis of breast cancer in samples with Her-2 positive breast cancer. These results implied that perhaps XBP1-HIF-1α would be involved in the metastatic process of both triple negative and Her-2 positive breast cancers. Notably, the expressions of MALAT1 in highly metastatic samples with TNBC were significantly higher than those in highly metastatic samples with Her-2 positive breast cancer (Figure 1), implying that XBP1-HIF1α pathway was activated to a relatively great extent in TNBC and might be play more important roles in the metastatic process of TNBC.

### Impacts of anaerobic conditions upon proliferation/migration abilities of triple negative and Her-2 positive breast cancer and XBP1-HIF1α

**Figure 2:**
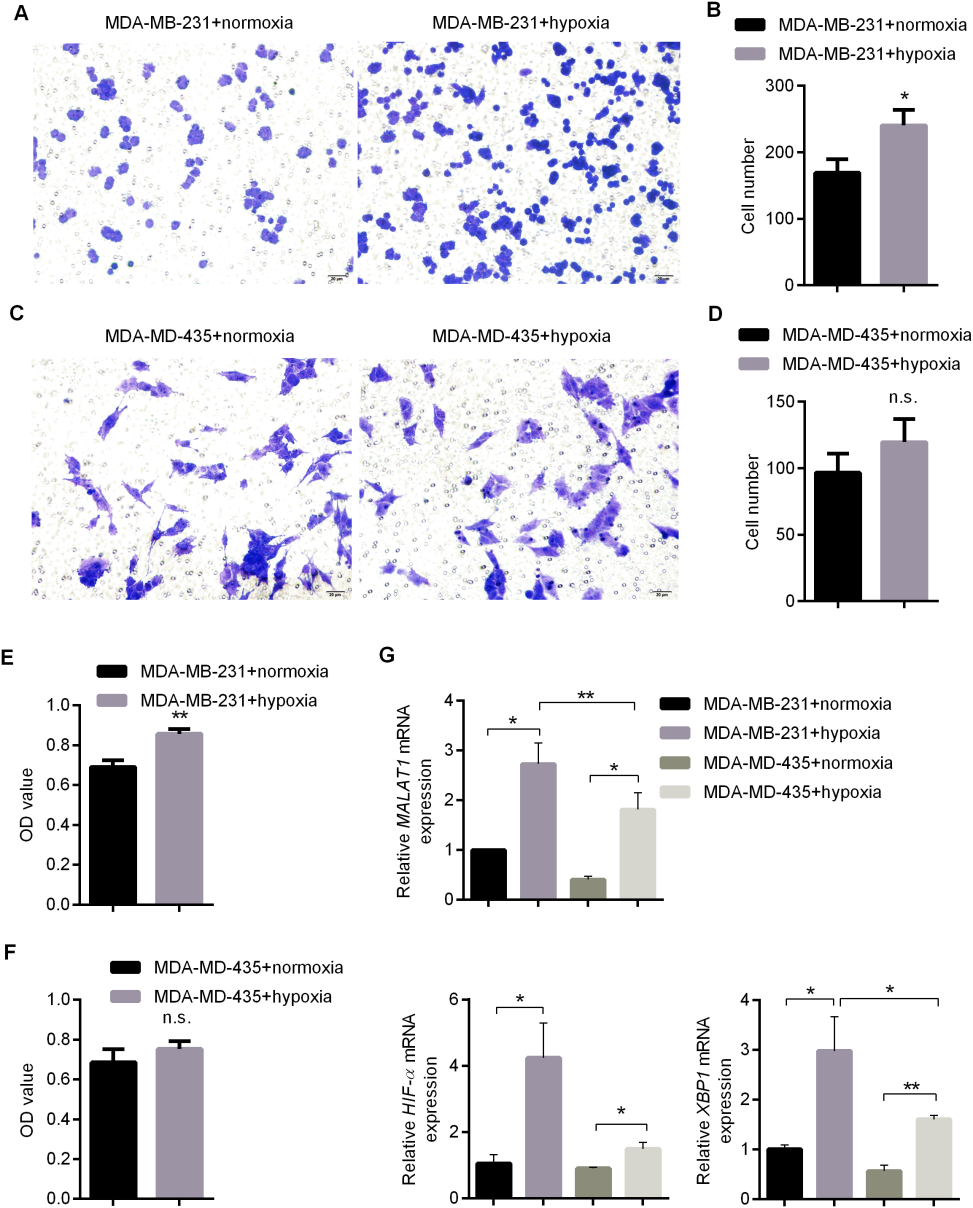
Impacts of Anaerobic Conditions upon Proliferation and Invasion Abilities of MDA-MB-231 and MDA-MD-435 **(A-B)** Invasion Ability of MDA-MB-231 Detected by Transwell Experiment and Related Statistical Result; **(C-D)** Invasion Ability of MDA-MD-435 Detected by Transwell Experiment and Related Statistical Result; **(E)** Viabiliy of MDA-MB-231 under Anaerobic or Normoxic Conditions after 48h of Culture Detected by CCK8 ; **(F)** Viability of MDA-MD-435 under Anaerobic or Normoxic Conditions after 48h of Culture Detected by CCK8; **(G)** Impacts of Anaerobic Conditions upon Expressions of MALAT1, HIF-1α and XBP1 Detected by qRT-PCR. ^*^, p<0.05; ^**^, p<0.01; n.s., no significance.

*in vivo*, anaerobic conditions were favorable for development and metastasis of breast cancer [20]. In previous studies, it has been demonstrated that under anaerobic conditions, XBP1-HIF1α promotes metastasis of TNBC [15]. Then, does MALAT1 act together with this pathway to promote the metastasis of TNBC? Does this pathway also stimulate metastasis of Her-2-positive breast cancer? To answer these questions, TNBC cell line MDA-MB-231 and Her-2 positive breast cancer cell line MDA-MD-435 were selected in this study, and cells were cultured under hypoxic conditions to simulate anaerobic conditions *in vivo*. First of all, the expression levels of ER, PR and Her-2 in both cell lines were detected by qRT-PCR. It was clarified that MDA-MB-231 was a breast cancer cell with low expressions of ER, PR and Her-2, while MDA-MD-435 was a breast cancer cell with low expressions of ER and PR but high expressions of Her-2 (Supplementary Figure 1). CCK8 and transwell experiments suggested that under anaerobic conditions, cell proliferation and invasion abilities of MDA-MB-231 were enhanced (Figure 2A-2F). However, the proliferation and invasion abilities of MDA-MD-435 were not significantly improved under anaerobic conditions (Figure 2A-2F). Subsequently, the changes to expressions of MALAT1, XBP1 and HIF1α in aforementioned two kinds of cells were detected by qRT-PCR under anaerobic conditions. Under anaerobic conditions, the expressions of XBP1 significantly increased in both MDA-MB-231 and MDA-MD-435, whereas XBP1 was activated much more significantly in MDA-MB-231 compared with MDA-MD-435 (Figure 2G). Similarly, the expressions of HIF1α significantly increased in both types of cells under anaerobic conditions, and XBP1 was activated more significantly in MDA-MB-231 than MDA-MD-435 (Figure 2G). It was particularly important that under anaerobic conditions, the expression level of MALAT1 was significantly higher in two types of cells (Figure 2G). These results indicated that under hypoxic culture conditions, proliferation and invasion abilities of breast cancer cells were strengthened. Besides, XBP1-HIF1α was activated. The enhancement and activation were more evident in triple-negative and Her-2 positive breast cancer cells. On the other hand, the expressions of MALAT1 increased under anaerobic conditions, which suggested that MALAT1 would play crucial roles in development and metastasis of breast cancer.

### MALAT1 is required for proliferation and invasion abilities of triple negative and Her-2 positive breast cancer cells

Based on the findings in the Section 3.3, hypoxic models were selected to study the roles of MALAT1 in MDA-MB-231, whereas the roles of MALAT1 in MDA-MD-435 were examined with normoxic models. At first, three siRNAs specific to MALAT1 were synthesized, and the expressions of MALAT1 in two kinds of cells were knockdown via transfection. To verify knockdown effects of siRNAs, the expressions of MALAT1 in two kinds of cells were detected by qRT-PCR after transfection, and the results revealed that siRNA1 can reduce the expressions of MALAT1 most stably and effectively (Supplementary Figure 2A). Therefore, siRNA1 was chosen as a tool for subsequent knockdown of MALAT1.

**Figure 3:**
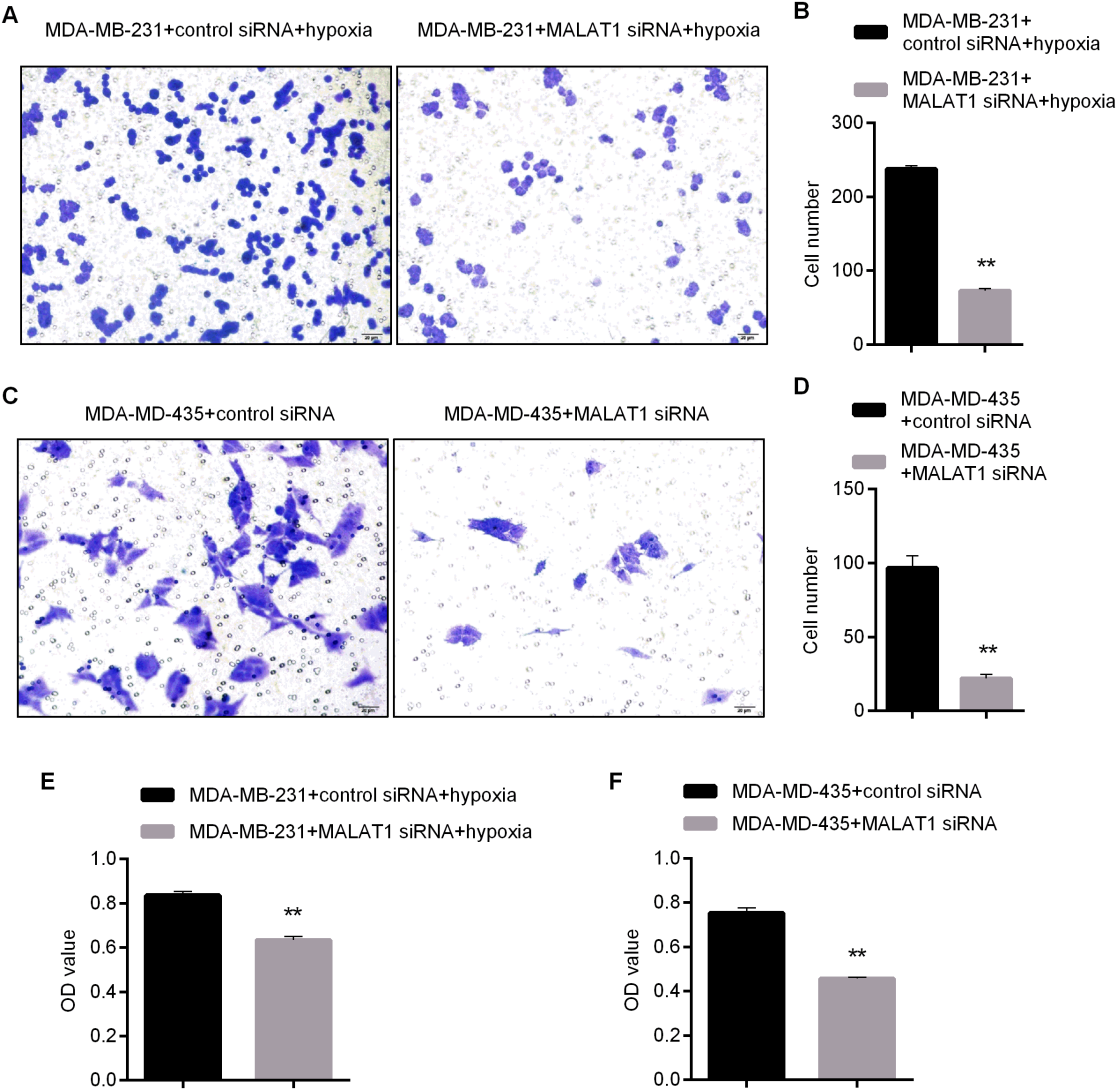
Impacts of MALAT1 Knockdown upon Proliferation and Invasion Abilities of MDA-MB-231 and MDA-MD-435 **(A-B)** Invasion Ability of MDA-MB-231 Detected by Transwell Assay after MALAT1 Knockdown and Related Statistical Results; **(C-D)** Invasion Ability of MDA-MD-435 Detected by Transwell Assay after MALAT1 Knockdown and Related Statistical Results; **(E)** Viability of MDA-MB-231 Detected by CCK8 after after MALAT1 Knockdown and 48h of Culture Later ; **(F)** Viability of MDA-MD-435 Detected after MALAT1 Knockdown and 48h of Culture under Normoxic Conditions. ^*^, p<0.05; ^**^, p<0.01.

Next, changes to proliferation and invasion abilities of the two cell lines were tested by CCK8 and transwell experiments after the knockdown of MALAT1. By knocking down MALAT1, it was effective for suppressing proliferation and invasion abilities of MDA-MB-231 under anaerobic conditions (Figure 3A, 3B, 3E). Under normoxic conditions, it was also effective for enhancing proliferation and invasion abilities of MDA-MD-435 by knocking down MALAT1 (Figure 3C, 3D, 3F). These results suggested that MALAT1 promoted proliferation and invasion of both kinds of breast cancer cells.

### MALAT1 promotes proliferation and invasion abilities of TNBC cells through XBP1-HIF1α pathway

**Figure 4:**
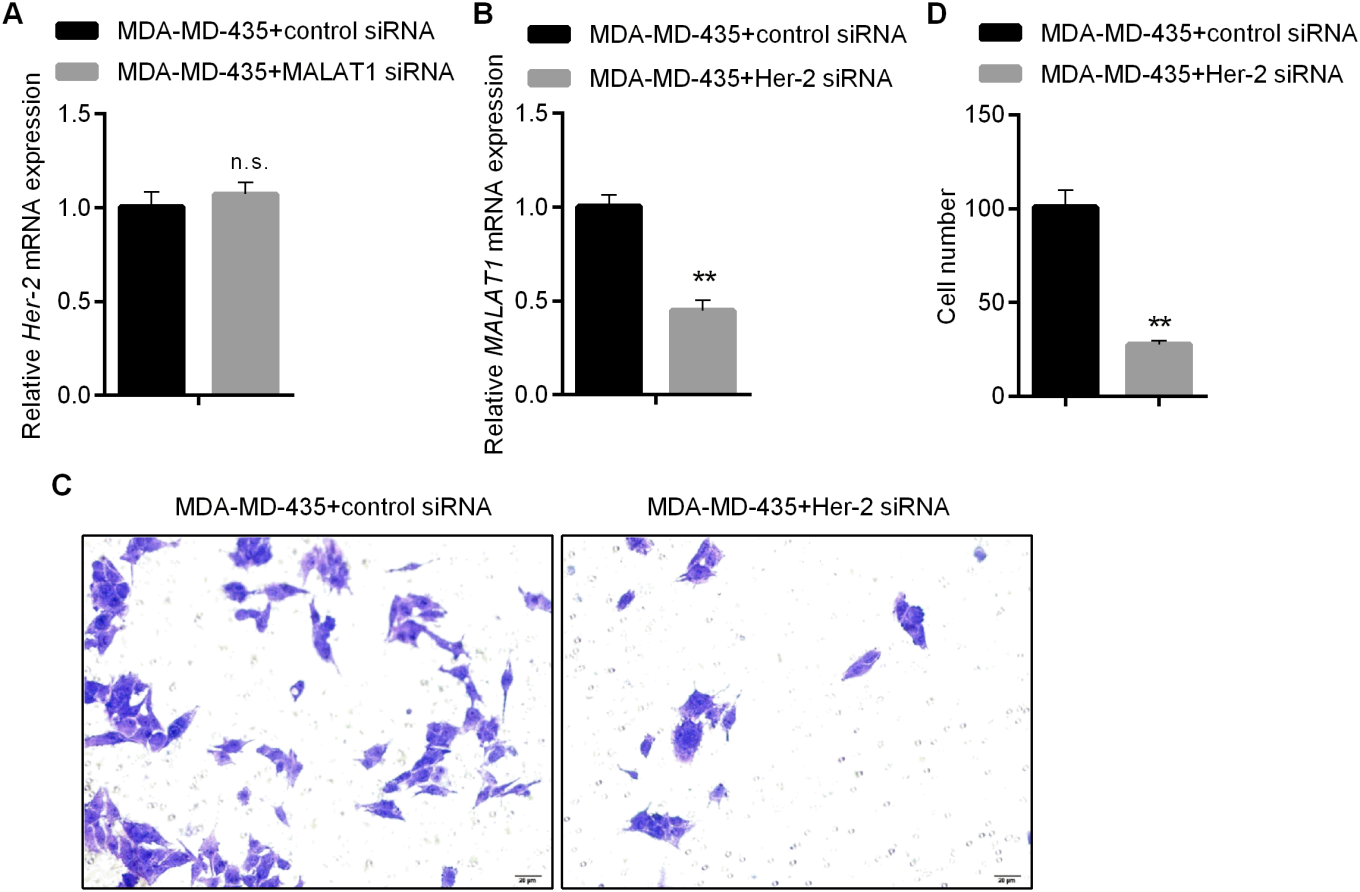
Involvement of MALAT1 in Regulating Proliferation and Invasion Abilities of Triple-negative Breast Cancer Cells by XBP1-HIF1α **(A)** Impacts of MALAT1 Knockdown upon Expressions of XBP1, HIF1α and MALAT1 Detected by qRT-PCR; **(B)** Impacts of XBP1 Knockdown upon Expressions ofMALAT1 and HIF1α Detected by qRT-PCR; **(C-D)** Invasion Ability of MDA-MB-231 Detected by Transwell Assay after XBP1 Knockdown and Related Statistical Results. ^*^, p<0.05; ^**^, p<0.01. n.s., no significance.

To explore whether MALAT1 regulates proliferation and invasion abilities of triple-negative breast cancer cells through XBP1-HIF1α, we firstly detected the expressions of XBP1 and HIF1α in MDA-MB-231 by qRT-PCR after the knockdown of MALAT1. As shown in Figure 4A, after the knockdown of MALAT1, no significant change happened to the expression levels of XBP1 and HIF1α. Hence, we further studied the impacts of XBP1 knockdown upon proliferation/invasion abilities of MDA-MB-231 and expressions of MALAT1. Through detection by qRT-PCR, siRNA1 that could knock down the expressions of XBP1 most effectively was selected from three siRNAs as a tool for subsequent research (Supplementary Figure 2B). qRT-PCR analysis revealed that knockdown of XBP1 could effectively reduce the expressions of HIF1α and MALAT1 (Figure 4B), which suggested that expressions of MALAT1 were regulated by XBP1-HIF1α. In addition, it was effective for suppressing proliferation and invasion abilities of MDA-MB-231 under anaerobic conditions by XBP1 knockdown (Figure 4C-4D). According to these results, MALAT1 promotes proliferation and invasion abilities of TNBC cells through XBP1-HIF1α.

### MALAT1 promotes proliferation and invasion abilities of Her-2 positive breast cancer cells

**Figure 5:**
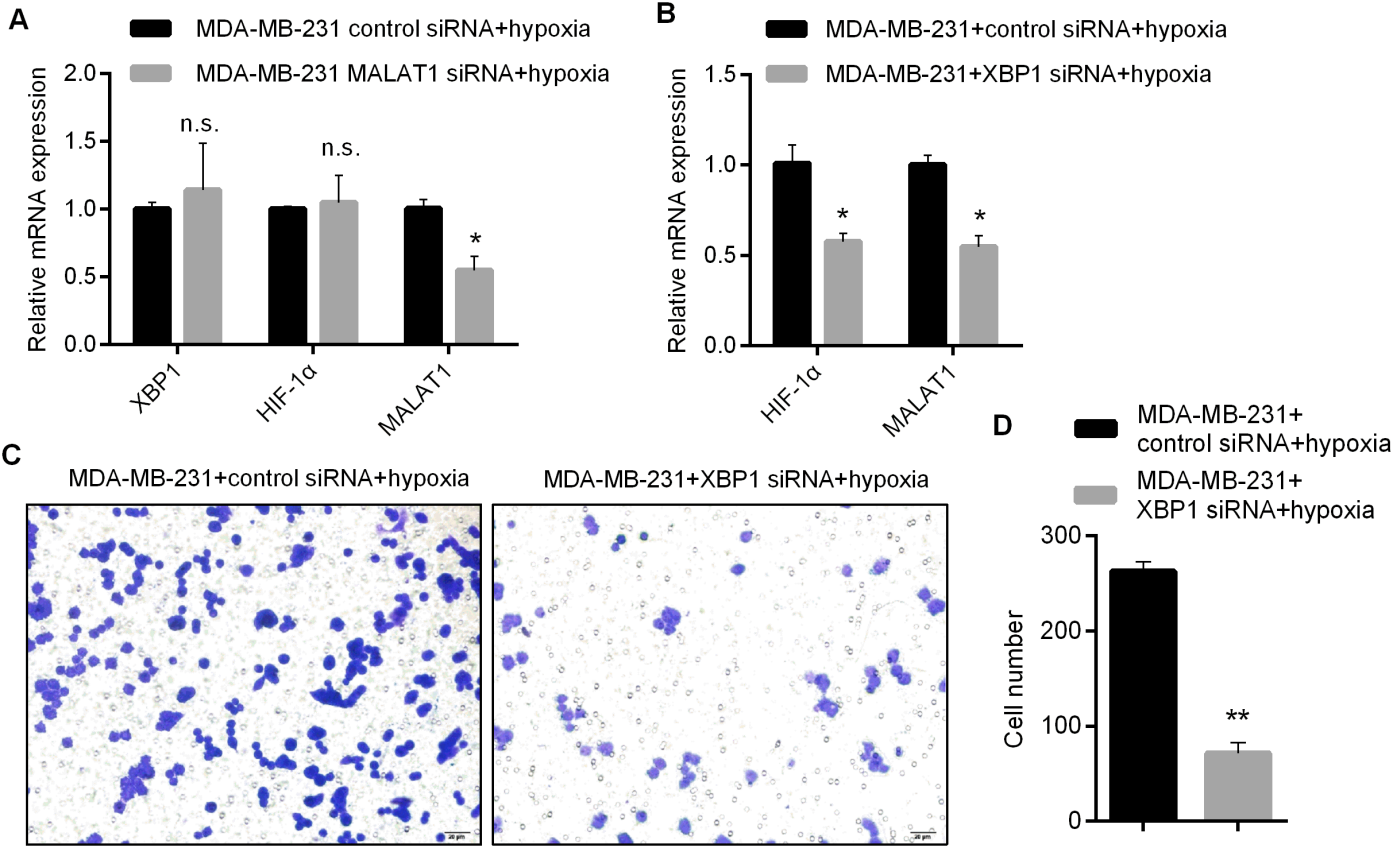
Regulating Proliferation and Invasion Abilities of HER2-positive Breast Cancer Cells by MALAT1 **(A)** Impacts of MALAT1 Knockdown upon Expressions of HER2 Detected by qRT-PCR; **(B)** Impacts of HER2 Knockdown upon Expressions of MALAT1 Detected by qRT-PCR; **(C-D)** Invasion Ability of MDA-MD-435 Detected by Transwell Assay after HER2 Knockdown and Related Statistical Results.^*^, p<0.05; ^**^, p<0.01. n.s., no significance.

To explore whether MALAT1 was involved in regulating proliferation and invasion abilities of Her-2 positive breast cancer cells via Her-2, we firstly detected the expressions of Her-2 in MDA-MD-435 by qRT-PCR after the knockdown of MALAT1. According to the results, no significant change occurred to the expression level of Her-2 after the knockdown of MALAT1 (Figure 5A). Therefore, we further explored the impacts of Her-2 knockdown upon proliferation/invasion abilities of MDA-MD-435 and the expression level of MALAT1. Through detection by qRT-PCR, siRNA1 that could knock down the expression of Her-2 most effectively was selected from three siRNAs as a tool for subsequent research (Supplementary Figure 2C). Her-2 knockdown was effective for downregulating the expression of MALAT1 (Figure 5B). Moreover, knockdown of Her-2 inhibited proliferation and invasion abilities of MDA-MD-435 under normoxic conditions (Figure 5C-5D). These results indicated that MALAT1, located in the downstream region of Her-2, was involved in promoting proliferation and invasion abilities of Her-2 positive breast cancer cells.

### Involvement of MALAT1 in regulating expressions of immune checkpoint genes in triple negative and Her-2 positive breast cancer cells

**Figure 6:**
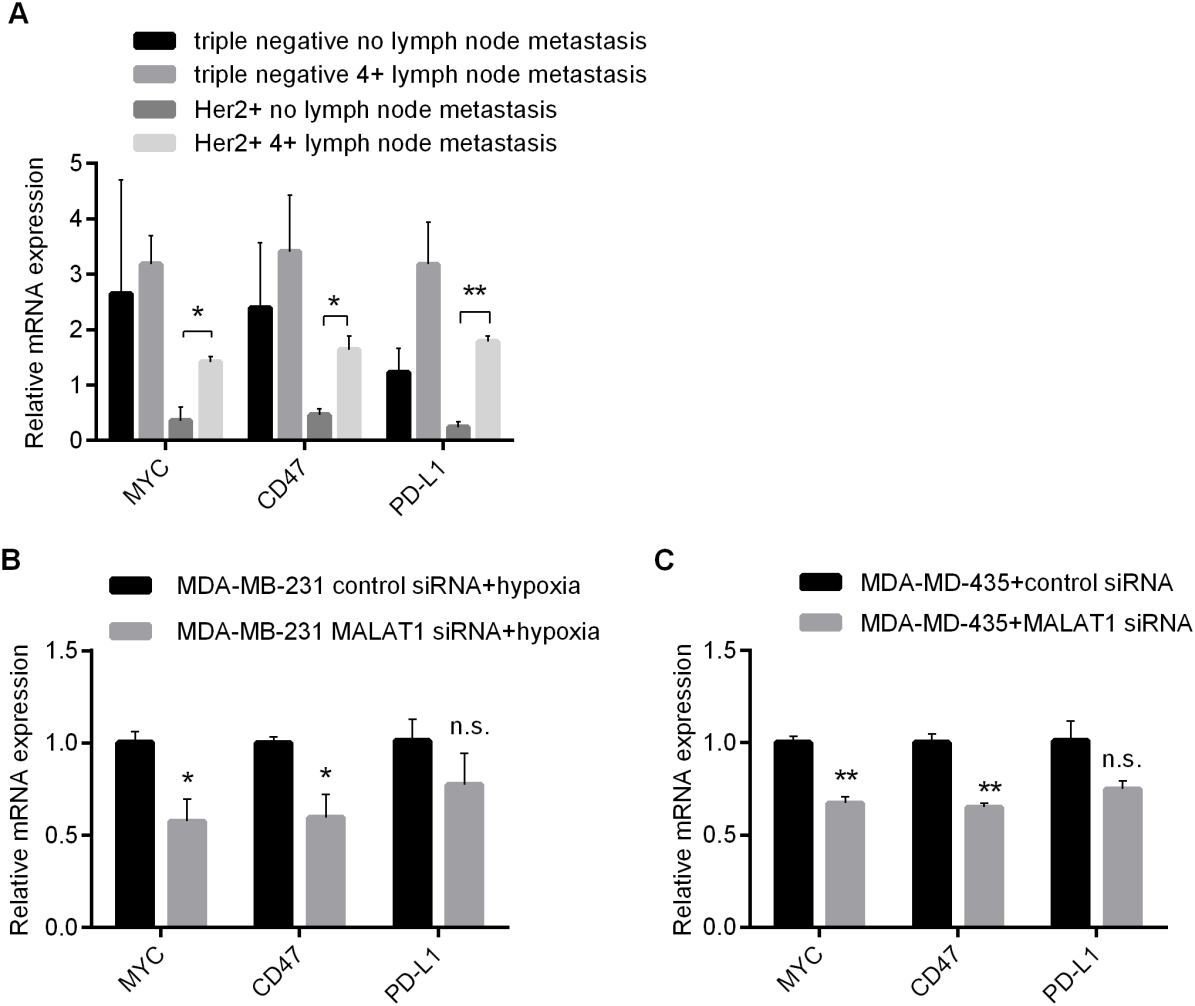
Involvement of MALAT1 in Regulating Expressions of Immune Checkpoint Genes in Above 2 Types of Breast Cancer Cells **(A)** Expressions of MYC, CD47 and PD-L1 in 2 Kinds of Breast Cancer without and with metastatic lymph nodes (≥4) Detected by qRT-PCR; **(B)** Impacts of MALAT1 Knockdown upon Expressions of MYC, CD47 and PD-L1 in MDA-MB-231 Detected by qRT-PCR; **(C)** Impacts of MALAT1 Knockdown upon Expressions of MYC, CD47 and PD-L1 in MDA-MD-435 Detected by qRT-PCR. ^*^, p<0.05; ^**^, p<0.01. n.s., no significance.

In addition to directly enhancing its own proliferation and migration abilities, MALAT1 could affect interactions between tumor cells and host immune environment. The expressions of MYC and its downstream immune checkpoint genes (CD47 and PD-L1) in samples with TNBC and Her-2 positive breast cancer were detected by qRT-PCR. The results suggested that the expressions of MYC, CD47 and PD-L1 didn't have any significant difference between highly metastatic and non-metastatic samples with TNBC. However, their expressions were much higher in samples with highly metastatic Her-2 positive breast cancer compared with other sampled without metastasis (Figure 6A). These results implied that MYC, CD47 and PD-L1 might be related to metastasis and relapse of Her-2 positive breast cancer. *In vitro* cell culture experiments revealed that the expression of MYC and CD47 decreassed significantly in MDA-MB-231 after the knockdown of MALAT1 under anaerobic conditions. Under normoxic conditions, the expressions were significantly downregulated in MDA-MD-435 after MALAT1 was knockdown (Figure 6C). Altogether, these results suggested that expressions of both MYC and CD47 were positively regulated by MALAT1 in triple negative and Her-2 positive breast cancers, which implied that MALAT1 might get involved in immune escape of breast cancer cells.

## DISCUSSION

It has been recently demonstrated that long non-coding RNAs are functional, and playing critical roles in genesis and development of tumors [21]. As a novel and representative lncRNAs, MALAT1 was found to be highly expressed in several kinds of cancer. In this study, the expressions of MALAT1 in samples with triple negative and Her-2 positive breast cancer, which are two kinds of breast cancer with the highest degree of malignancy and the poorest prognosis, were detected. The analysis of small samples suggested that the expressions of MALAT1 could be negatively correlated to prognosis and survival, but positively related to both metastasis and relapse of both kinds of cancer. Hence, we preliminarily considered that MALAT1 was possibly a common marker for prognosis of both types of cancer. Coincidence with our studies, Jadaliha and his colledges have reported that MALAT1 would function as a driver gene before metastasis of ER-negative breast cancer (triple negative and Her-2 positive), and in patients without metastatic lymph nodes, the high expressions of MALAT1 suggested high risks of metastasis and relapse as well as poorer prognosis [7].

With varying expressions in different molecular subtypes of breast cancer, MALAT1 has distinct mechanisms of action. According to the research findings in this study, MALAT1 shows much higher expressions in samples with TNBC than samples with Her-2 positive breast cancer. Besides, its expressions are higher in MDA-MB-231 than those in MDA-MD-435. From the perspective of the mechanisms, our findings demonstrated that XBP1 and HIF-1α are upstream driver genes of MALAT1 in TNBC cells, while Her-2 is an upstream driver gene of MALAT1 in Her-2 positive breast cancer cells. In both types of breast cancer, different signaling pathways promote tumorgenesis and progression through a common effector molecule, which is known as MALAT1. On the other hand, it has been reported by other researchers that MALAT1 promotes the development of breast cancer by getting involved in transcriptional regulation [10] and alternative splicing [11], therefore it may be considered as a core signaling molecule for promoting proliferation and migration of breast cancer.

The vast deregulation of lncRNAs in cancers, as well as their involvement in various cancer related pathways implies that lncRNAs can be used as specific targets for breast cancer treatment. According to their different roles in breast cancer development, many lncRNAs-based target therapies can be designed. Antisense oligonucleotides (ASOs), siRNAs, as well as viral vectors that contain short hairpin RNA (shRNAs) can be used for modification of oncogenic lncRNAs expression [22]. The result of ASO-mediated inhibition of lncRNAs in animal model has been promising. Recently, a study published in NEJM has indicated that ASO-mediated silencing of MALAT1 in a metastasis luminal B breast cancer model can result in a cystic, poorly metastatic phenotype that closely mimicked those that arose in animals with genetic deletion of Malat1 [8]. Notably, we have demonstrated in our study that genetic interventions with MALAT1 are effective for inhibiting proliferation and invasion abilities of triple-negative and Her-2 positive breast cancer cells. Therefore, MALAT1 is not only a potential target for treating Luminal B breast cancer [8], but would be also applicable to triple-negative and Her-2 positive breast cancers. Compared with other types of breast cancer, there is a lack of specific targeted drugs and treatment options for TNBC, which is a type of highly malignant breast cancer characterized by high relapse rate within 3 years after surgeries and low post-relapse survival rate [23]. Thus, MALAT1 is important for treating TNBC as a potential therapeutic target. Although the effect of MALAT1 silencing in TNBC and Her-2 positive breast cancer remains to be further verified in animal models, the results of above-mentioned experiments would pave the way for breast cancer clinical trials of MALAT1 in molecular targeted therapies.

According to the theory of immunotherapy, the interaction between the host immune system and tumor cells was regulated by a series of genes that act as immune checkpoints. Breast cancer cells can evade from the host immune surveillance by upregulating their own immune checkpoint genes [24]. CD47 prevents antigen presentation by suppressing phagocytosis of macrophage and dendritic cells which are both required for host innate immunity [18], while PD-L1 regulates the acquired immunocompetence of hosts by suppressing immune toxicity of T cells [19]. These immune checkpoint genes have been demonstrated to be highly expressed in many cancer cells. In our study, it was found that like MYC, which is a well-known oncogene, MALAT1 might be also involved in regulating immune checkpoint genes such as CD47. In future studies, efforts should be paid to verify whether MALAT1 really gets involved in regulation of host immunocompetence and whether genetic interventions with MALAT1 is likely to strengthen the effectiveness of host immune systems for killing tumor cells.

## MATERIALS AND METHODS

### Statistics of clinical information and sample collection

Information of clinical samples was collected from the tumor sample bank of frozen tissues in Zhejiang Cancer Hospital. Five cases with TNBC who had no metastatic lymph node, five cases with TNBC who had 4 or more metastatic lymph nodes, five cases with Her-2 positive breast cancer who had no metastatic lymph node, and five cases with Her-2 positive breast cancer that had 4 or more metastatic lymph nodes were selected. All these cases have been registered in the sample library since 2010. Details of cases are shown in Supplementary Table 1. The breast cancer samples were collected on the days of initial surgeries, so all of them were fresh. After they were frozen with liquid nitrogen, they were transferred and stored at at -80°C. The overall survival (OS) was statistically analyzed in the period from the time of initial diagnosis to the time of last outpatient service or follow-up visit. RFS was statistically investigated from the time of initial diagnosis to the time of identifying relapse by further diagnosis or from the time of initial diagnosis to the last outpatient service or follow-up visit (relapse-free).

### Cell culture and plasmid transfection

Both cell lines MDA-MB-231 and MDA-MD-435 were purchased from Shanghai Fuxiang Biological Technology Co., Ltd, China. Cells were cultured on RPMI 1640 Medium (Hyclone) under normoxic conditions. SiRNAs (small interfering RNAs) of cells in the logarithmic phase (confluence: 50% to 80%) were transfected. The sequences of siRNAs are shown in Supplementary Table 2. Transfection steps shall conform to the User Manual of Lipofectamine™ 2000 Reagent (Invitrogen). Some cells were taken 48 hours after the transfection to detect effects of gene knockdown by qRT-PCR. The remained cells were further cultured for 48 hours under normoxic (21% O_2_, 5% CO_2_) or anaerobic (1% O_2_, 5% CO_2_) conditions. Cells were collected for CCK8 and qRT-PCR detection.

### Detection of cell proliferation by CCK8

Cell proliferation was detected according to the User Manual of “Reagent Kit for Detecting Cell Proliferation and Cytotoxicity (Beyotime)”. In brief, cells which had been transfected for 48 hours or non-transfected cells were chosen and further cultured for 48 hours under normoxic or anaerobic conditions. 10μL CCK8 was added to each well of cells, cultured for 4 hours at 37°C. The optical density (OD) of each well was measured by an enzyme-labelling instrument.

### Detecting invasion abilities of cells by transwell experiment

Cells which had been transfected for 48 hours or non-transfected cells were further cultured for 48 hours under normoxic or anaerobic conditions. After pancreatic digestion, the cell suspension was collected. Matrigel (Invitrogen) was thawed on the ice. The serum-free culture medium was mixed with Matrigel at 7 to 1 (50 μL and 50 μg Matrigel/well were added to each compartment. After the mixing, the mixture was dripped into the chamber at 50μL through each well in the middle of chambers and placed at 37°C for 1 to 2 hours. After Matrigel was coagulated, cell medium was added on the lower layer of 24-well culture plate and placed inside chambers. Then, treated cell suspension was added to the chambers (5×10^5^/well). The culture media inside the chambers were discarded after they were placed there for about 20 hours, and washed by PBS (phosphate buffered saline). Matrigel and cells on the upper chamber surface were rubbed away with soaked cotton swabs. The cells which invaded into gel were fixed by methanol. The cells were stained with 0.1 Sigma, observed and photographed with a microscope.

### RNA Extraction and analysis of gene transcription level by qRT-PCR

Cells were collected and centrifugally precipitated. The tissue samples were sheared into small scraps, and total RNA was extracted by Trizol (Invitrogen). Purity and concentration of RNA were determined based on the OD260/280 ratio. The total RNA was reversely transcribed according to the User Manual of the Reagent Kit for Reverse Transcription (Fermentas). RT-PCR should be performed in line with the User Manual of the Reagent Kit for Real-time PCR (TransGen). Sequences of PCR primers are shown in Supplementary Table 3. The expression level of MALAT1 in all clinical samples was normalized together with β-actin, which was reckoned as internal control. Relative expression levels of genes in cell samples were measured by differential CT.

### Data analysis

All data were analyzed by SPSS 23.0 (software for data analysis) and Graphpad prism 6.0 (mapping software). The differences in expressions of certain gene between two groups were estimated by performing Student's t test. Correlations between expressions of MALAT1 and prognostic factors were estimated based on Pearson's correlation coefficients. The multivariate analysis was performed through stepwise multivariate regression. Two-sided tests were conducted, and the differences would be deemed to be significant when the value of P was lower than 0.05.

## SUPPLEMENTARY MATERIALS FIGURES AND TABLES



## References

[1] Siegel RL, Miller KD, Jemal A (2017). Cancer Statistics, 2017. CA Cancer J Clin.

[2] Fan L, Strasser-Weippl K, Li JJ, St Louis J, Finkelstein DM, Yu KD, Chen WQ, Shao ZM, Goss PE (2014). Breast cancer in China. Lancet Oncol.

[3] Goldhirsch A, Wood WC, Coates AS, Gelber RD, Thurlimann B, HJ; Senn (2011). Panel members. Strategies for subtypes--dealing with the diversity of breast cancer: highlights of the St. Gallen International Expert Consensus on the Primary Therapy of Early Breast Cancer.

[4] Cameron D, Piccart-Gebhart MJ, Gelber RD, Procter M, Goldhirsch A, de Azambuja E, Castro G, Untch M, Smith I, Gianni L, Baselga J, Al-Sakaff N, Lauer S (2017). 11 years' follow-up of trastuzumab after adjuvant chemotherapy in HER2-positive early breast cancer: final analysis of the HERceptin Adjuvant (HERA) trial. Lancet.

[5] Vikram R, Ramachandran R, Abdul KS (2014). Functional significance of long non-coding RNAs in breast cancer. Breast Cancer.

[6] Jin C, Yan B, Lu Q, Lin Y, Ma L (2016). Reciprocal regulation of Hsa-miR-1 and long noncoding RNA MALAT1 promotes triple-negative breast cancer development. Tumour Biol.

[7] Jadaliha M, Zong X, Malakar P, Ray T, Singh DK, Freier SM, Jensen T, Prasanth SG, Karni R, Ray PS, Prasanth KV (2016). Functional and prognostic significance of long non-coding RNA MALAT1 as a metastasis driver in ER negative lymph node negative breast cancer. Oncotarget.

[8] Mendell JT (2016). Targeting a long noncoding RNA in breast cancer. N Engl J Med.

[9] Dong Y, Liang G, Yuan B, Yang C, Gao R, Zhou X (2015). MALAT1 promotes the proliferation and metastasis of osteosarcoma cells by activating the PI3K/Akt pathway. Tumour Biol.

[10] Hirata H, Hinoda Y, Shahryari V, Deng G, Nakajima K, Tabatabai ZL, Ishii N, Dahiya R (2015). Long noncoding RNA MALAT1 promotes aggressive renal cell carcinoma through Ezh2 and interacts with miR-205. Cancer Res.

[11] Tripathi V, Ellis JD, Shen Z, Song DY, Pan Q, Watt AT, Freier SM, Bennett CF, Sharma A, Bubulya PA, Blencowe BJ, Prasanth SG, Prasanth KV (2010). The nuclear-retained noncoding RNA MALAT1 regulates alternative splicing by modulating SR splicing factor phosphorylation. Mol Cell.

[12] Fan Y, Shen B, Tan M, Mu X, Qin Y, Zhang F, Liu Y (2014). TGF-beta-induced upregulation of malat1 promotes bladder cancer metastasis by associating with suz12. Clin Cancer Res.

[13] Dittrich A, Gautrey H, Browell D, Tyson-Capper A (2014). The HER2 signaling network in breast cancer--like a spider in its web. J Mammary Gland Biol Neoplasia.

[14] Wu XS, Wang XA, Wu WG, Hu YP, Li ML, Ding Q, Weng H, Shu YJ, Liu TY, Jiang L, Cao Y, Bao RF, Mu JS (2014). MALAT1 promotes the proliferation and metastasis of gallbladder cancer cells by activating the ERK/MAPK pathway. Cancer Biol Ther.

[15] Chen X, Iliopoulos D, Zhang Q, Tang Q, Greenblatt MB, Hatziapostolou M, Lim E, Tam WL, Ni M, Chen Y, Mai J, Shen H, Hu DZ (2014). XBP1 promotes triple-negative breast cancer by controlling the HIF1alpha pathway. Nature.

[16] Salle-Lefort S, Miard S, Nolin MA, Boivin L, Pare ME, Debigare R, Picard F (2016). Hypoxia upregulates Malat1 expression through a CaMKK/AMPK/HIF-1alpha axis. Int J Oncol.

[17] Topalian SL, Drake CG, Pardoll DM (2012). Targeting the PD-1/B7-H1(PD-L1) pathway to activate anti-tumor immunity. Curr Opin Immunol.

[18] Jaiswal S, Jamieson CH, Pang WW, Park CY, Chao MP, Majeti R, Traver D, van Rooijen N, Weissman IL (2009). CD47 is upregulated on circulating hematopoietic stem cells and leukemia cells to avoid phagocytosis. Cell.

[19] Casey SC, Tong L, Li Y, Do R, Walz S, Fitzgerald KN, Gouw AM, Baylot V, Gutgemann I, Eilers M, Felsher DW (2016). MYC regulates the antitumor immune response through CD47 and PD-L1. Science.

[20] Schito L, Rey S, Tafani M, Zhang H, Wong CC, Russo A, Russo MA, Semenza GL (2012). Hypoxia-inducible factor 1-dependent expression of platelet-derived growth factor B promotes lymphatic metastasis of hypoxic breast cancer cells. Proc Natl Acad Sci U S A.

[21] Li L, Chang HY (2014). Physiological roles of long noncoding RNAs: insight from knockout mice. Trends Cell Biol.

[22] Soudyab M, Iranpour M, Ghafouri-Fard S (2016). The role of long non-coding RNAs in breast cancer. Arch Iran Med.

[23] Kumar P, Aggarwal R (2016). An overview of triple-negative breast cancer. Arch Gynecol Obstet.

[24] Criscitiello C, Esposito A, Gelao L, Fumagalli L, Locatelli M, Minchella I, Adamoli L, Goldhirsch A, Curigliano G (2014). Immune approaches to the treatment of breast cancer, around the corner?. Breast Cancer Res.

