# Supplementary material for: Roles of MALAT1 in development and migration of triple negative and Her-2 positive breast cancer
Source: Oncotarget. 2017 Dec 18;9(2):2255–67. doi: 10.18632/oncotarget.23370 (PMC5788637; doi:10.18632/oncotarget.23370)
Supplement: Supplementary file 1 [file oncotarget-09-2255-s001.pdf]

Roles of MALAT1 in development and migration of triple negative and Her-2 positive breast cancer

SUPPLEMENTARY MATERIALS

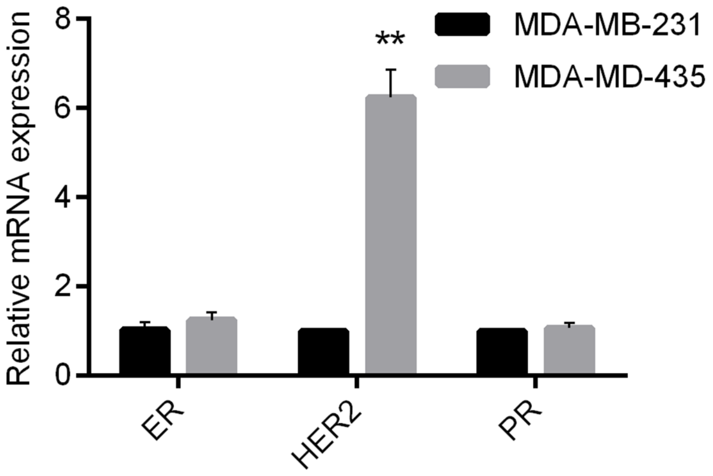

Supplementary Figure 1: Expressions of ER, PR and Her-2 in MDA-MB-231 and MDA-MD-435 Detected by qRT-PCR. **, $p<0.01$.

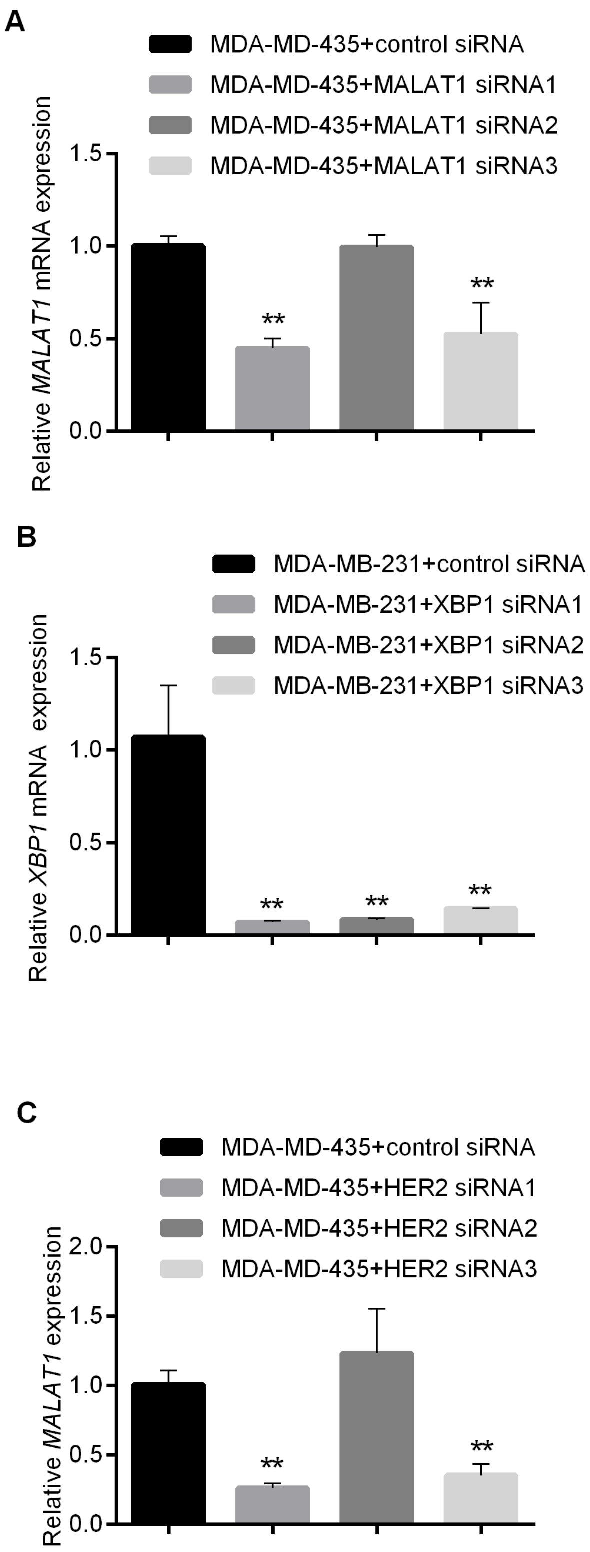

Supplementary Figure 2: Interference Effects of **(A)** MALAT1 siRNA, **(B)** XBP1 siRNA and **(C)** HER2 siRNA in MDA-MB-231 and MDA-MD-435 Detected by qRT-PCR. **, p<0.01.

Supplementary Table 1: Comparison of patient's related information and prognostic factors

NO.	Tumor type	First visit age	Pathological type	Clinical examination revealed the maximum diameter of the tumor(cT)/cm	Lymph node staging	pTNM pathological staging	Number of metastatic lymph nodes	OS/ months	RFS/ months
1	triple negative	44	Invasive medullary carcinoma	2.5	0	IIA	0	74	74
2	triple negative	63	Invasive ductal carcinoma	1.5	0	I	0	73	73
3	triple negative	56	Invasive ductal carcinoma	2.3	0	IIA	0	73	73
4	triple negative	39	Invasive ductal carcinoma	1.9	0	I	0	70	70
5	triple negative	46	Invasive ductal carcinoma	2.2	0	IIA	0	64	64
6	triple negative	43	Invasive ductal carcinoma	4.3	2	IIIA	7	81	81
7	triple negative	47	Invasive ductal carcinoma	2.2	2	IIIA	8	74	74
8	triple negative	82	Invasive ductal carcinoma	5	3	IIIC	17	23	6
9	triple negative	27	Invasive ductal carcinoma	2.5	2	IIB	4	51	21
10	triple negative	55	Invasive ductal carcinoma, part of tumor is metaplastic carcinoma	2.3	2	IIB	4	84	84
11	Her-2 positive	60	Invasive ductal carcinoma	3	0	IIA	0	88	88
12	Her-2 positive	41	Invasive ductal carcinoma with giving priority to intraductal carcinoma	6.5	0	IIB	0	86	38
13	Her-2 positive	43	Invasive ductal carcinoma	1.2	0	I	0	72	72
14	Her-2 positive	43	Invasive ductal carcinoma with giving priority to intraductal carcinoma	4	0	I	0	70	70
15	Her-2 positive	71	Invasive ductal carcinoma	4.5	0	IIA	0	72	72
16	Her-2 positive	52	Invasive ductal carcinoma	3	2	IIIA	4	75	8
17	Her-2 positive	53	Invasive ductal carcinoma	1.5	2	IIIA	4	69	69
18	Her-2 positive	66	Invasive ductal carcinoma	5	2	IIIA	6	24	16
19	Her-2 positive	49	Invasive ductal carcinoma	6	3	IIIC	17	52	42
20	Her-2 positive	59	Invasive ductal carcinoma	2.7	3	IIIC	21	30	18

Supplementary Table 2: SiRNA sequence information

Name		Sequence
MALAT1- siRNA1	sense	GATCCATAATCGGTTTCAAGG
	Anti-sense	TTGAAACCGATTATGGATCAT
MALAT1- siRNA2	sense	CACAGGGAAAGCGAGTGGTTGGTAA
	Anti-sense	TTACCAACCACTCGCTTTCCCTGTG
MALAT1- siRNA3	sense	GGGCTTCTCTTAACATTTATT
	Anti-sense	TAAATGTTAAGAGAAGCCCTT
XBP1- siRNA1	sense	ACAGCAAGTGGTAGATTTATT
	Anti-sense	TAAATCTACCACTTGCTGTTT
XBP1- siRNA2	sense	AGCTAATGTGGTAGTGAAATT
	Anti-sense	TTTCACTACCACATTAGCTTT
XBP1- siRNA3	sense	CACCCTGAATTCATTGTCTTT
	Anti-sense	AGACAATGAATTCAGGGTGTT
Her2- siRNA1	sense	CCTGTTCTCCGATGTGTAAGG
	Anti-sense	TTACACATCGGAGAACAGGGG
Her2- siRNA2	sense	AGAGTGATGTGTGGAGTTATG
	Anti-sense	TAACTCCACACATCACTCTGG
Her2- siRNA3	sense	GGGGCTGGCTCCGATGTATTT
	Anti-sense	ATACATCGGAGCCAGCCCCTT

Supplementary Table 3: q-PCR primer sequence information

Name	Sequence
Homo PR-F	ATCCTGCCTCTCAATCACG
Homo PR-R	TTATCTTTAGAGCGGGCGG
hER-F	CCAGTACCAATGACAAGGGA
hER-R	CCACCTTTCATCATTCCCAC
hHER2-F	AGACAGAGTACCATGCAGAT
hHER2-R	GATCCCATCGTAAGGTTT
ACTB-F	GATGAGATTGGCATGGCTTT
ACTB-R	GTCACCTTCACCGTTCCAGT
hCD47-F	GGCACAAAACACTACTGAAG
hCD47-R	GAGACAGCATCACTCTTATC
hMALAT1-F	AAAGCAAGGTCTCCCCACAAG
hMALAT1-R	GGTCTGTGCAAGATCAAAAGGCA
hPD-L1-F	GTAAGACCACCACCACCAAT
hPD-L1-R	TTCACATCCATCATTCTCCC
hHIF-1α-F	GGACAGTACAGGATGCTTGC
hHIF-1α-R	CGTGCTGAATAATACCACTC
hc-MYC-F	AAAACAATGAAAAGGCCC
hc-MYC-R	CACAAGAGTTCCGTAGCTGT
hXBP1-F	CAGACTGCCAGAGATCGAAA
hXBP1-R	CTGGCCTCACTTCATTCCC
